# In Vivo Facilitated Diffusion Model

**DOI:** 10.1371/journal.pone.0053956

**Published:** 2013-01-18

**Authors:** Maximilian Bauer, Ralf Metzler

**Affiliations:** 1 Institute of Physics and Astronomy, Potsdam University, Potsdam-Golm, Germany; 2 Physics Department, Technical University of Munich, Garching, Germany; 3 Physics Department, Tampere University of Technology, Tampere, Finland; Weizmann Institute of Science, Israel

## Abstract

Under dilute in vitro conditions transcription factors rapidly locate their target sequence on DNA by using the facilitated diffusion mechanism. However, whether this strategy of alternating between three-dimensional bulk diffusion and one-dimensional sliding along the DNA contour is still beneficial in the crowded interior of cells is highly disputed. Here we use a simple model for the bacterial genome inside the cell and present a semi-analytical model for the in vivo target search of transcription factors within the facilitated diffusion framework. Without having to resort to extensive simulations we determine the mean search time of a lac repressor in a living *E. coli* cell by including parameters deduced from experimental measurements. The results agree very well with experimental findings, and thus the facilitated diffusion picture emerges as a quantitative approach to gene regulation in living bacteria cells. Furthermore we see that the search time is not very sensitive to the parameters characterizing the DNA configuration and that the cell seems to operate very close to optimal conditions for target localization. Local searches as implied by the colocalization mechanism are only found to mildly accelerate the mean search time within our model.

## Introduction

Transcription factors (*TFs*) are able to locate and bind their target sequence on DNA at surprisingly high rates. This became clear when in 1970 it was measured that *in vitro* the lac repressor associates with the operator at a rate of 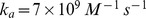 [1]. This is about two orders of magnitude faster than a rate calculated with the well-known Smoluchowski formula for three-dimensional diffusion control [2]. The results obtained in the *in vitro* experiments by Riggs et al. and by Winter et al. were successfully explained with the by now classical facilitated diffusion model, introduced by Berg, von Hippel and co-workers [3], [4]: the TF alternates between three-dimensional diffusion through the bulk solution and sliding along the DNA contour which can be considered as one-dimensional diffusion. While a large majority of subsequent reformulations of this target search problem are based on this facilitated diffusion model [5]–[8], there are also critical reviews focusing on limitations of the traditional model [9], [10].

Even if it is accepted by most of the scientists that *in vitro* TFs perform facilitated diffusion to find their targets, there is a vivid debate on whether this mechanism indeed plays a role *in vivo*. The interest in this long-standing topic was boosted by the development of new experimental techniques, namely single-molecule assays studying DNA-binding proteins, or more generally the diffusion of proteins within cells [11]–[18]. After finding indirect evidence some years ago, Elf and coworkers recently demonstrated that the lac repressor does display facilitated diffusion in live *Escherichia coli (E. coli)* cells [19], [20].

Thus it is important to study how the present facilitated diffusion models need to be translated to the *in vivo* situation. In comparison to the dilute situation studied *in vitro* the most important changes are: the influence of the confinement to the cell body or the nucleoid and the compactified DNA conformation, and the impact of the presence of many large biomolecules. The latter, which is often referred to as macromolecular crowding has two major effects: the equilibrium for DNA-binding proteins is shifted favoring the associated state and the diffusion in the cytoplasm is slowed down [21], [22]. There is an on-going debate whether this reduced diffusion is still Brownian, following experimental evidence that for larger molecules such as mRNA [23], [24] or lipid granules [25] the motion follows the laws of anomalous diffusion [26], [27]. Indeed, there are indications that particles of the size of several tens of kilo Daltons exhibit anomalous diffusion [28], [29]. In what follows we model TFs in the bulk by normal Brownian diffusion and point at potential implications of anomalous diffusion in the conclusions.

We note that theoretical work on facilitated diffusion *in vivo* has also been reported by Mirny and coworkers as well as by Koslover and coworkers [9], [30]. A different approach for the situation in living cells, based on a fractal organization of the chromatin in the nucleus, showed that also in eukaryotes facilitated diffusion can be beneficial [31].

With respect to the impact of the cell's finite size Foffano et al. recently studied the influence of (an-)isotropic confinement on the facilitated diffusion process for rather short DNA chains [32]. To build a theoretical model for facilitated diffusion on the entire genome in living cells we shortly review what is known about the organization of the bacterial DNA [33]. The emerging general consensus points at a distinct separation of the genome into connected subunits, that may be dynamic. Using atomic force microscopy the size of structural units of the *E. coli* chromosome was studied, finding units of size 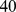 nm and 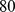 nm [34]. By means of two complementary approaches the average size of the structural domains was measured to be 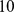 kilobasepairs (kbp) [35]. Romantsov et al. studied the structure with fluorescence correlation spectroscopy, yielding units of size 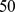 kbp with a diameter of 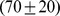 nm [36]. Chromosome conformation capture carbon copy(5C) was used to determine a three-dimensional model of the *Caulobacter* genome [37]. For the same bacterium Viollier et al. determined that the location of genes on the chromosome map correlates linearly with its position along the cell's long axis [38].

Based on these experimental observations several models for the DNA structure in bacterial cells have been proposed: entropy is spotted to be the main driver of chromosome segregation, and ring polymers are used to model the bacterial chromosome [39], [40]. Buenemann and Lenz showed that a geometric model based on a self-avoiding random walk (SAW) is sufficient to explain the linear positioning of loci along the cell's longest axis [41]. Finally, the chromosomal structure and, in particular, the accurate positioning of loci was proposed as resulting from regulatory interactions [42], [43].

In this paper we survey if it is possible to extend our previous generalized facilitated diffusion model [44] to the *in vivo* situation and compare the results with the ones obtained by Koslover et al. [30]. Therefore in the following section we detail how we obtain a coarse-grained model for the bacterial genome and state our semi-analytical model for the search process. Then the general theory will be applied to the specific case of a lac repressor in an *E. coli* cell, and we favorably compare our results with related experimental measurements [19]. Finally we conclude our findings and give an outlook on future research directions.

## Theory

The quantity we investigate is the average time a TF needs to find a target sequence in a living bacterial cell after starting at a random position within the cell. In principle it is possible to apply our previous generalized diffusion model using rescaled rates, lengths and diffusion constants to account for the crowded *in vivo* environment [44]. However, for parameters typical for the interior of cells the effective contact radius between TF and DNA is larger than the average distance between neighboring DNA segments. Consequently a direct translation is not possible.

Moreover, as we will see below, already the simpler one-state model of facilitated diffusion is sufficient to obtain a fairly good estimate of the experimental results without any further free parameters. Thus we do not distinguish between search and recognition states of the TF-DNA complex [5]. Intersegmental jumps and/or transfers [6], [8], [16], [45] of TFs between DNA segments, that are close-by in the embedding space but distant when measured in the chemical coordinate along the genome, are to some extent indirectly included in terms of re-attachment to the DNA within one of the geometric subunits of the chromosome. In future studies these effects could be explicitly included to refine the model.

Our approach is based on the general picture of the facilitated diffusion mechanism: the TF diffuses three-dimensionally through the bulk solution until it encounters a stretch of DNA to which it can bind. Then a sliding motion along the DNA contour is possible, during which the TF probes for the target. If the target is not found, the TF will dissociate from the chain after a certain time span and resume its 3D-diffusion through the cell until the next binding event. This scheme continues until the target is found. The major difference to the dilute *in vitro* situation lies in the DNA conformation which is heavily influenced by the confinement to the cell volume or the nucleoid volume: As the contour length of (the typically circular) bacterial DNA is about three orders of magnitude larger than the longest cell axis in which it is placed, there is clearly a need to compact it. To proceed we present our model for the compacted genome.

### Model for the compacted genome

Without dwelling on details to which extent nucleoid-structuring proteins and/or supercoiling is responsible for DNA compaction in bacterial cells, we adapt the model of Buenemann and Lenz and assume that the DNA is assembled structurally into spheres (‘blobs’) containing one loop each [41]. Thus, the whole genome is modeled as a closed SAW of these uniformly large blobs on a lattice representing the nucleoid volume (here we diverge from ref. [41], where the full cell volume was taken). To mimic the cylindrical shape of the nucleoid one of the cuboid lattice's edges is taken to be longer than the other two of equal length.

**Figure 1:**
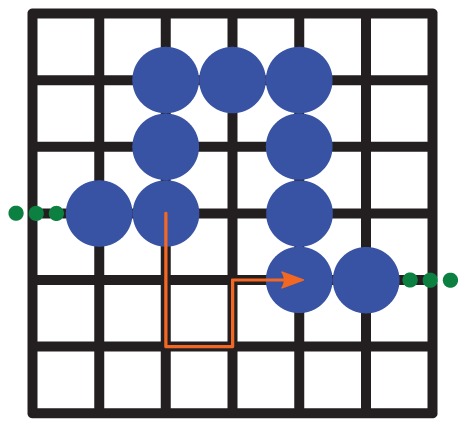
Two-dimensional schematic of the DNA conformation. The circles denote single DNA blobs. The lattice spacing is twice the blob radius: 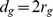. A part of an exemplary search trajectory is depicted by the arrow.

The key quantities are the blobs' radius of gyration 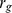 and the number of basepairs within a blob, 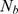. While the latter parameter determines how many blobs make up the DNA, since the number of bps on the DNA is a fixed parameter, the first one effectively determines the lattice size (see figure 1).

To obtain individual DNA conformations we follow a routine similar to the one described in ref. [41]: as a starting point we use a closed loop of minimal extension which touches both end faces along the longest cell axis. Then the chain is elongated by inserting hooks at random positions until it reaches the desired length (due to the form of the algorithm only chains with an even number of blobs are considered). Only elongation steps which yield a conformation within the nucleoid volume are executed. Afterwards the genome is equilibrated in the following manner: we randomly choose one of the three transformation types of the MOS algorithm [46]. Then it is checked if the resulting conformation is still an SAW, otherwise the old conformation is kept. Finally only attempts are counted in which the SAW is still confined to the nucleoid volume. This is repeated 100,000 times for each individual model genome.

Afterwards the resulting DNA conformations are centered on a larger lattice representing the full cell volume and remain unchanged during the subsequent simulation of the target search process. This approach is affirmed by recent results that DNA dynamics only have little effect on target search rates [30]. For the sake of simplicity we assign the target to be in a blob in the middle of the DNA.

### Target search process

**Figure 2:**
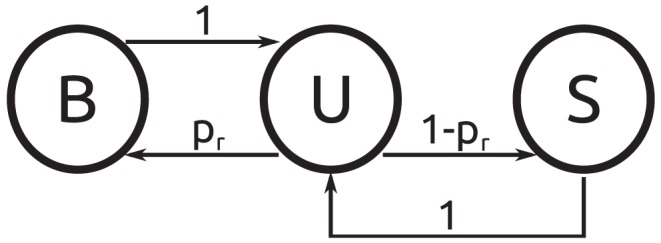
Schematic of the microscopic events within a blob (without target). B denotes a bound TF, and U an unbound TF within a blob. Finally, S represents a searching TF which is currently not in a blob.

The TF is assumed to start its search at a random position in the cell volume and its motion is modeled as a random walk on the effective lattice (fig. 1), during which we keep track of how often sites containing a blob are passed. The search process is schematically depicted in fig. 2. The TF starts its search diffusing in 3D (S-state). With certainty (probability 1) after some time it will encounter a blob, which it enters in its unbound state (U). We first study the case where this blob does not contain the target DNA. Based on the microscopic model be outlined below, we assign a probability 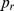 that the TF will bind to the DNA within this blob. If so it changes to the B-state. As there is no target to be found on the DNA, after some time the TF will dissociate and return to the unbound U-state. With probability 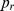 it can bind again, or it may leave the blob (with probability 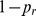) and start a new random walk on the lattice (S-state). The same procedure will take place when subsequent blobs are encountered.

**Figure 3:**
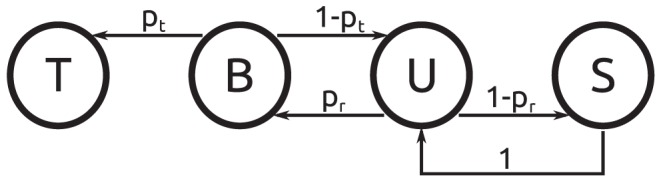
Schematic of the microscopic events within the target blob. Same notation as in the previous figure. Additionally, T denotes a TF which has found the target.

A qualitatively new event occurs when the site containing the target DNA is encountered for the first time. Now the tendency to quit the corresponding blob competes with the probability to find the target. For this reason, in general several encounters with the target blob are necessary. The corresponding scheme is depicted in figure 3: Once again after entering the blob in the unbound U-state, with probability 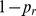 not a single binding event takes place. However, if the TF binds to DNA (with probability 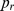), subsequently with probability 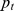 the target will be found (T-state) before dissociating. If the target is not found and the TF dissociates, again with probability 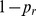, the blob is left. Otherwise (with probability 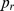) a new chance to find the target while being bound is opened up. As in the simpler scheme without target, a new random walk (S-state) is started on a neighboring site if the blob is left. To proceed we relate the probabilities 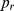 and 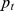 to microscopic quantities and determine the time steps of the individual processes, before calculating the typical search time for the target.

### Microscopic model

To determine 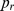, that is the probability to bind to DNA after entering a blob or after dissociation from the DNA within the blob we employ the approximation that locally the DNA can be treated as a random coil [3], [44]. Thus we have to solve the diffusion equation for an initially homogeneous probability distribution within a sphere of radius 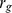. Inside this sphere nonspecific association to a basepair on the DNA occurs with the constant, intrinsic rate 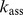 (in units of 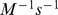). We introduce a second concentric sphere of radius 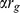 whose surface is absorbing, modeling the TFs leaving the domain of the blob. Thus, the dimensionless quantity 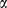 measures (in units of 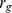) where the blob's area of influence ends, see below and Supporting Information S1. The corresponding problem is solved in the SI S1, yielding the binding probability
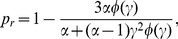
(1)with the dimensionless quantity 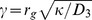. Here 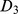 denotes the 3D-diffusion constant, and 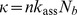. Moreover, 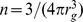 represents the density of DNA within the coil. In Eq. 1 we also introduced the auxiliary function 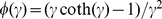 [47].

Note that 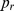 is a monotonic function of 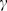. Keeping the values of 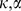 and 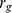 fixed, for decreasing, yet finite values of 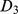 the probability to escape the blob (which is given by 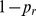) becomes smaller, as in this case the TF moves slower and spends more time within the blob, where it can be caught by a stretch of DNA. Exactly at 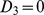 one obtains 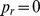, an apparent paradox. However, while it is true that an immobile TF is unable to leave a blob, the converse argument that the TF will bind to DNA with certainty is not obvious, as binding requires the motion of a TF towards DNA within the blob. Because this complementarity is implicitly assumed in the present model, it only yields meaningful results for finite values of 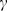. Only this situation will be considered in the following.

If binding occurs, the average time this takes is given by a somewhat complicated formula for arbitrary values of 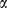 (see SI S1). Here we report the simpler result for the special case 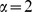. This case is of interest, as in the numerical evaluation we use the value 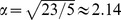, see below.
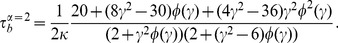
(2)Conversely, the average time it takes the TF to leave the blob is
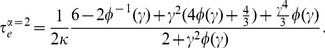
(3)While diffusing in 3D, a single random walk step on average takes 
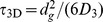
. Once the TF binds non-specifically to the blob containing the target, the probability to find the target before dissociating can be found by considering a one-dimensional diffusion problem. We assume that the target is located in the middle of the corresponding blob. Then we consider a DNA stretch of length 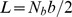 with the target at one end. Here 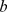 denotes the size of a basepair, 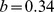 nm.

Due to the DNA's coiled conformation within a blob, we use the standard assumption that the first binding event occurs at a random position on the DNA and that dissociation and reassociation positions are completely uncorrelated, see for example [48]. Formally this implies that the TF initially is uniformly distributed on the DNA along which it diffuses with the diffusion constant 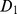. The TF can leave the DNA with the dissociation rate 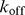. We furthermore assume that the other extremity of the DNA acts as a reflecting boundary [48], possibly due to compacting proteins that obstruct further 1D-diffusion at this position. The calculation detailed in the SI S1 yields:
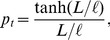
(4)


with 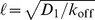, which denotes a typical distance covered sliding on DNA before dissociating. If the target is found, the conditional time this successful event takes on average, reads
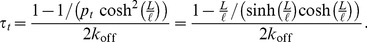
(5)


However, an unsuccessful event implies that the DNA is (on average) left after the time span 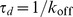. Inspection of Eq. (5) shows that in the limit 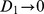, i.e. when TFs are (nearly) incapable of sliding, 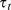 approaches the finite value 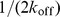, which is at first sight a surprising result. However, in this limit the probability to reach the target as given by Eq. (4) approaches zero, ensuring that meaningful results are obtained. It should be stressed that our model only allows target detection via sliding, and not via direct detection solely through three-dimensional diffusion.

### Mean search time

To determine the mean time it takes to find the target at first we specify how often the “loop” of binding and unbinding events (B and U in figures 1 and 2) is executed during an encounter with a blob. In all the blobs without the target this happens on average 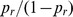 times. As one loop lasts 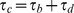 the average time that is spent within a blob is 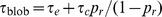.

In the blob containing the target, the average number of binding and unbinding loops is 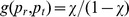, where 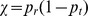. Note that the number of executed loops in blobs without target is the special case 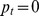 of the general case, 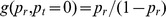. In the same sense figure 2 can be considered a special case of figure 3. The combined probability to find the target before leaving the blob reads 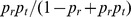, consequently the probability for a failed attempt is 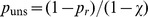. Thus, a successful event during which the target is found, on average takes 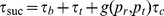, and an unsuccessful one 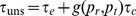.

The mean total search time can be dissected into three contributions: first, the mean time the TF needs to arrive at the target blob for the first time. Then the mean time it takes to return to the target after an unsuccessful search event. The latter has to be multiplied with the average number of failed attempts. Finally the average time it takes to successfully bind the target at the corresponding blob has to be added.

To quantify this model two parameter pairs from the random walk simulation are needed as inputs: the mean number of steps it takes to encounter the target blob for the first time 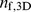 after starting at a random position within the cell and how many blobs without target are encountered during this time, 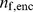. Furthermore we determine the mean number of steps and blob-encounters in a random walk starting on a site next to the target blob: 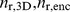 and ending in the target blob. Altogether the mean total search time reads:
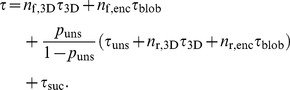
(6)This formula is the main result of our study, which will be discussed quantitatively for the case of the lac repressor in an *E. coli* cell.

## Results

As input parameters for our TF search model in a living cell we use data deduced from experimental studies. For the DNA configuration we use two parameter sets for the blob size and the number 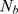 of basepairs within a blob: (a) 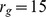 nm and 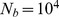 [35], [41] and (b) 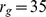 nm and 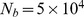 [36]. The volume of the nucleoid can be approximated as a cylinder of diameter 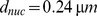 and length 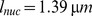 [39]. We use a cuboid with edge lengths 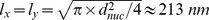 and 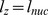. This corresponds to nucleoid lattices of size 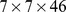 and 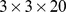. As the *E.coli* genome consists of 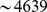 kbps, we compose a closed SAW consisting of (a) 464 blobs and (b) 92 blobs, respectively. For the parameter sets we create three and five sample conformations. The total cell volume can be approximated as a cylinder with 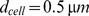 and length 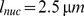 [39]. Accordingly, we use embracing lattices of size 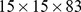 and 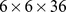 to mimic the full cell volume. Besides, we employ 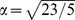 in order to obtain the correct asymptotic behavior for small values of 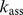 as detailed in the SI S1 and we use 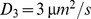 and 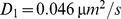 [19]. The results of the random walk simulation are summarized in table 1.

**Table 1:** Simulation results.

Set	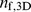	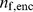	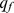	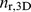	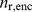	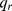
a	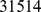	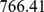	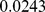	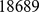	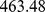	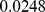
b	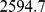	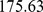	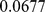	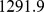	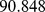	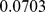

Simulation results for parameter sets a and b.

A first inspection of the values of 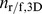 and 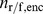 shows that the ones obtained with parameter set a are approximately one order of magnitude larger than the ones obtained with set b. This is clear as set a corresponds to a finer model of the DNA, in which the respective value of 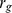 is smaller. Next, we consider the ratios 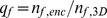 and 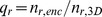, that is the fractions of sites containing a blob encountered during a trajectory. The results are very close to the total fraction of sites that are occupied by a blob: for parameter set a, this is: 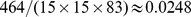 and for b: 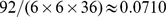. This and the fact that the values for the first encounter and for the returning trajectories are similar, support the statement that the TF experiences an effective medium through which it diffuses [30]. If we only consider the mean search times, this medium is mainly characterized by the mean DNA density within the cell.

### Non-monotonic behavior

**Figure 4:**
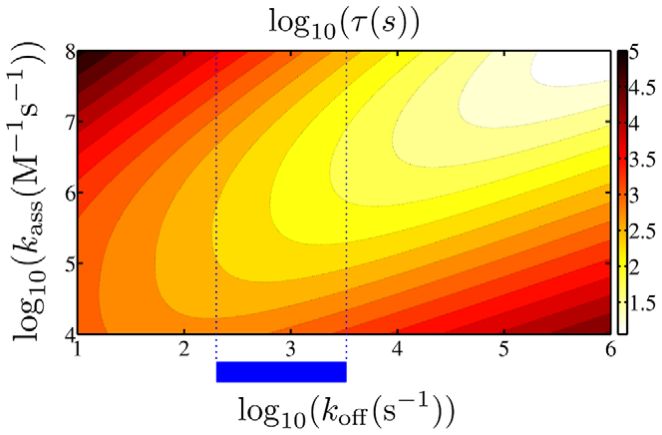
Mean search time. The mean search time is plotted as a function of the dissociation rate 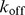 and the association rate 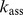 (using parameter set a). The blue bar and the blue dotted lines denote the range of 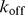 which is biologically relevant [19].

In figure 4 the mean search time averaged over the ensembles with parameter set a is shown as a function of the association rate 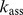 and the dissociation rate 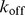. We find a non-monotonic dependence both on the association and the dissociation rate typical for facilitated diffusion models: for a fixed value of 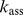 there exists a value of 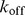 that minimizes the search time. This minimal value decreases if both rates are increased while keeping them at a constant ratio.

**Figure 5:**
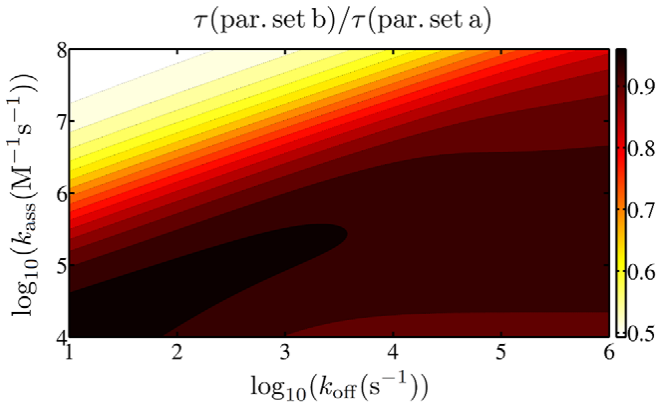
Difference between the two parameter sets. The plot shows the ratio of the mean search time obtained with parameter set b with the ones obtained with set a.

In figure 5 the ratio of the search time obtained with parameter set b with the search time obtained with parameter set a is plotted for the same range as in figure 4. Even though set b always yields slightly smaller search times, the results are very similar, especially in the range usually studied in experiments, as we will see below. Therefore in the following we solely consider results obtained with set a. In the SI S1 we moreover show that the approach to use an ensemble average to obtain the mean search time is justified as the scatter between data obtained with individual conformations is negligible (see figure S1). Only at very low values of 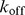, when the TF spends considerable time in the non-specifically bound state, the individual conformation does play a role.

We saw that for fixed values of 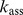, there exists an optimal of 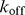, for which the target localization occurs fastest. It is insightful to study whether a living *E. coli* cell operates close to this point.

### Comparison to experimental results

**Figure 6:**
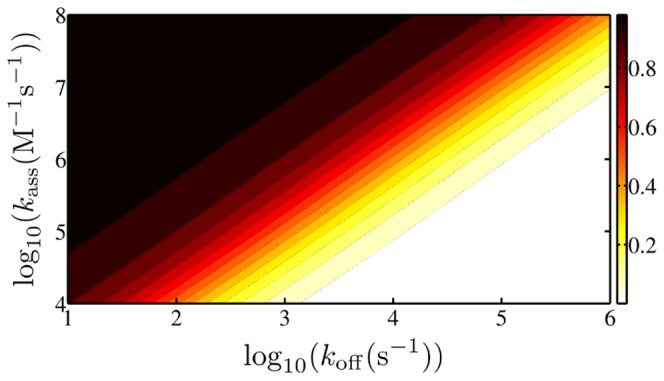
Bound fraction of time. The fraction of time during which the TF is non-specifically bound is shown (using parameter set a).

We choose the rates according to the results of Xie and coworkers [19]: they measured that the lac repressor spends 87% of the total time non-specifically bound and determined the residence time on DNA 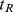 to be in the range
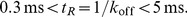
(7)To incorporate these values, we calculate the fraction of time, 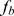, that the TF spends non-specifically bound. This is obtained from Eq. 6 by only considering the terms involving 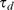 and 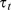. The result is plotted in figure 6, again as a function of dissociation and association rate.

**Figure 7:**
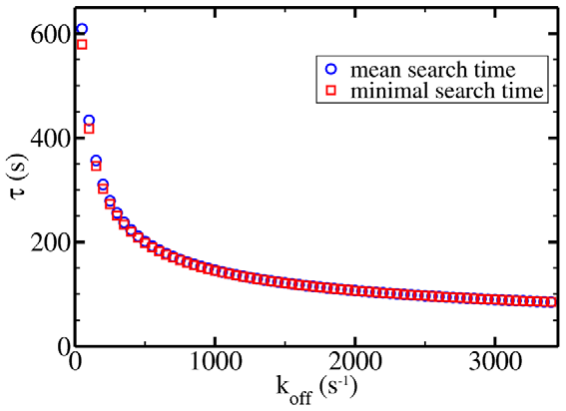
Mean search time and minimal search time. The mean search time and the minimal search time (with appropriately chosen 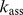) are plotted as a function of the dissociation rate at parameters relevant for the interior of living cells.

We see that contour lines of a constant fraction appear as straight lines in this log-log-plot. A numerical analysis yields that the condition 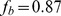 is fulfilled for
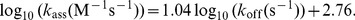
(8)The observation that the slope of this curve is (nearly) unity, reflects the fact that specifying the bound fraction of time is equivalent to specifying the equilibrium binding constant which is simply given by the ratio of 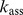 and 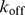. We plug Eq. 8 into our model and plot the resulting mean search time as a function of the single residual parameter 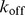 in figure 7 in the range given by Eq. 7. Additionally, in figure 7 we plot the minimal search time in this regime which is obtained by choosing the optimal value of 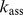.

In both cases we obtain a monotonically decreasing function of 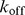. Most interestingly, the values obtained in this biologically relevant parameter regime are only marginally larger than the optimal ones. At 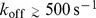 the two data sets nearly lie on top of each other. This means that within our model an *E. coli* cell seems to operate quite close to conditions, which are optimal for target localization. At 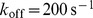, which was used in the discussion in ref. [30], we obtain 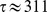 s. This is approximately 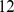
% below the experimental result 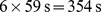 [19], implying a very favorable agreement.

### Local searches

**Figure 8:**
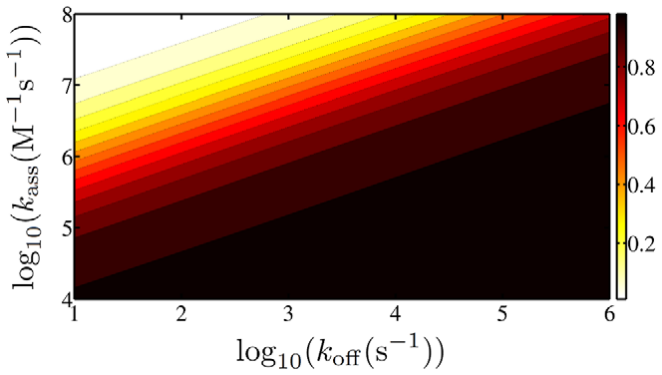
Acceleration due to local searches. The ratio of the time needed in a local search with the one in a global search (with parameter set a) is shown.

There is some evidence that many TFs are produced close to their target positions, a phenomenon called colocalization [14], [49]. These local searches would obviously be faster than a global search starting at a random position within the cell. To quantify this in figure 8 we plot how many percent of the total search time is still needed to find the target if the TF starts its search in the target blob while all other parameters remain unchanged. In mathematical terms this corresponds to omitting the terms in the first line of Eq. 6. We see that only for relatively large values of 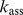 an appreciable acceleration is obtained for local searches. This is clear as large values of the association rate imply that all the blobs encountered en route act as traps slowing down the transport. Interestingly, in the regime typical for the interior of cells the acceleration is of little amount. This can also be interpreted in the more general context of “geometry-controlled kinetics”, see the works of Bénichou and coworkers [50], [51]. These authors showed that for non-compact exploration of space - as is the case in the present model - the initial position of a searching particle has little influence.

## Discussion

We analyzed the facilitated diffusion mechanism in a living cell using a coarse-grained model of the bacterial genome. Just like in dilute *in vitro* systems there is a non-monotonic dependence both on the dissociation rate and the association rate of TFs from and to DNA. The respective optimal conditions mark a trade-off between spending too much time on DNA where the motion is rather slow, but the target can be found, and spending too much time in the cytoplasm where the motion is faster, but the TF is insensitive to the target.

When calculating the mean search time as an input from our random walk simulation we solely use the mean number of steps taken and the number of blobs encountered during the trajectory. This corresponds to treating the nucleoid body as an effective medium through which the TF diffuses, which agrees with the observations made by Koslover et al. that within a short time span the TF starts an effective diffusive motion [30]. Accordingly, we see that the exact values of the parameters describing the DNA conformation have only little effect on our results. Only the fact that there is an effective medium characterized by the DNA density matters.

Calibrating our results with the experimental observation that the TF spends 87% of the time non-specifically bound [19], we obtain search times that only slightly underestimate the experimentally known results. In a previous study we showed that the introduction of a search and a recognition state in order to resolve the speed-stability paradox slows down the search [44]. Thus, a refined model taking this effect into account could yield a result even closer to the experimental one.

Most importantly, within our model the results in the biologically relevant regime of dissociation rates are quite close to the ones minimizing the search time, indicating that living *E. coli* cells function near conditions optimal for TF target location.

Our results for the mean search times are similar to those obtained by Koslover et al. [30]. However, in their model for *in vivo* facilitated diffusion they distribute the DNA over the entire cell volume and assume a random coil configuration. If one were confining the DNA to the smaller nucleoid volume, the effective DNA-TF contact radius in that model would then become smaller than the average distance between DNA segments. Besides, our model is less idealized. In that sense our current approach has the advantage that the DNA is realistically confined to the nucleoid volume, and based on input parameters deduced from experimental studies we also obtain mean search times, that are very close to experimental *in vivo* values. Moreover, our model offers the advantage that in future studies additional information may be deduced, for example, by studying the underlying probability densities of 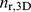, 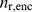, etc., in addition to their mean values determined here.

### Colocalization effects

Comparing the mean search times for TFs starting at a random position in the cell volume with those TFs that already start close to the target, we only observe a minor acceleration. This is due to the fact that most of the search time is spent returning to the target blob after a failed attempt to find the target. For a wide range of parameters the first encounter with the target blob only represents a small fraction of the whole search time. Leaving the picture of mean values for the search time of an ensemble of TFs, on the level of single trajectories immediate returns to the target blob are indeed possible and thus may lead to search times much shorter than the average search time. Such scenarios may in fact be relevant for biological cells.

Should observations of anomalous diffusion for TFs in the cytoplasm of living cells be substantiated, the effect of colocalization should become significantly more pronounced, if the nature of the exploration of space is compact [50], [51]: subdiffusion implies an increased occupation probability near the initial position [23], [52], [53], and thus increases the likelihood for successful TF-DNA binding after repeated attempts. In that sense subdiffusion may even be beneficial for molecular processes in living cells, as argued recently [52], [54], [55].

We believe that this relatively simple model for facilitated diffusion in vivo will instigate new experiments and more detailed theories, to ultimately obtain a full understanding of bacterial gene regulation.

## Supporting Information

Figure S1
**Ratio of the mean search times obtained with individual conformations with the respective ensemble averaged mean search time at** 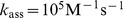 **.**
(EPS)

Supporting Information S1 **In this supporting information we detail the explicit calculations which are beyond the scope of the main text.**
(PDF)
