# Supplementary material for: In Vivo Facilitated Diffusion Model
Source: PLoS One. 2013 Jan 18;8(1):e53956. doi: 10.1371/journal.pone.0053956 (PMC3548819; doi:10.1371/journal.pone.0053956)
Supplement: Supporting Information S1 — In this supporting information we detail the explicit calculations which are beyond the scope of the main text. (PDF) [file pone.0053956.s002.pdf]

Supporting information S1: In vivo facilitated diffusion model

Maximilian Bauer[1,2,*] and Ralf Metzler[1,3,†]

[1] Institute for Physics & Astronomy, Potsdam University, 14476 Potsdam-Golm, Germany
[2] Physics Department, Technical University of Munich, 85747 Garching, Germany
[3] Physics Department, Tampere University of Technology, FI-33101, Finland

In this supporting information we detail the explicit calculations which are beyond the scope of the main text.

I. MICROSCOPIC MODEL

A. Association probability

To relate p_r to the non-specific association rate $k_{\rm ass}$ per base pair (in units of $M^{-1}s^{-1}$), we solve the following diffusion equation for the TF's probability $c(\mathbf{r},t)$ to be at position $\mathbf{r}$ at time t:

$$\frac{\partial c(\mathbf{r},t)}{\partial t} = \begin{cases} D_3\Delta c(\mathbf{r},t) - \kappa c(\mathbf{r},t), & \text{for } 0<r<r_g \\ D_3\Delta c(\mathbf{r},t), & \text{for } r_g<r<r_2 \end{cases}, \tag{S1}$$

with $\kappa = nk_{\rm ass}N_b$, where n denotes the density of DNA and N_b the number of basepairs within the blob. D_3 denotes the 3D-diffusion constant and r_g the blob's radius of gyration. The differential equation is subject to the initial condition

$$c(\mathbf{r},t=0) = \begin{cases} c_0 = 3/(4\pi r_g^3), & \text{for } 0<r<r_g \\ 0, & \text{for } r_g<r<r_2 \end{cases}, \tag{S2}$$

and the boundary condition $c(r=r_2,t)=0$. Thus, r_2 represents a cutoff-radius at which the TF is assumed to have definitely left the domain of the blob. We use $n=c_0$ as we study the situation where one TF is in the blob containing one DNA chain.

We define the Laplace transform $f(u)$ of a function $f(t)$ through:

$$f(u) = \int_0^\infty f(t)\exp(-ut)dt. \tag{S3}$$

In Laplace space the differential equation S1 reads:

$$uc(u,r) = \begin{cases} c_0 + D_3\Delta c(\mathbf{r},u) - \kappa c(\mathbf{r},u), & \text{for } 0<r<r_g \\ D_3\Delta c(\mathbf{r},u), & \text{for } r_g<r<r_2 \end{cases}, \tag{S4}$$

From its solution the flux out of the outer sphere $j_{\rm out}(u)$ and the binding flux $j_{\rm bind}(u)$ in the inner sphere can be obtained via:

$$j_{\rm out}(u) = -4\pi r_2^2 D_3 \left.\frac{\partial c(u,r)}{\partial r}\right|_{r=r_2}, \tag{S5}$$

and

$$j_{\rm bind}(u) = 4\pi\kappa\int_0^{r_g} dr\, r^2 c(u,r). \tag{S6}$$

We obtain

$$j_{\rm out}(u) = \frac{3}{r_g^3q_1^3}\frac{r_2q_2}{\sinh(q_2\delta r)}\frac{q_1r_g\coth(q_1r_g)-1}{\coth(q_1r_g)+\frac{q_2}{q_1}\coth(q_2\delta r)}, \tag{S7}$$

and furthermore

$$j_{\rm bind}(u) = \frac{3}{r_g^3q_1^3}\frac{\kappa}{u+\kappa}\left[\frac{r_g^3q_1^3}{3} - \frac{(q_1r_g\coth(q_1r_g)-1)(1+r_gq_2\coth(q_2\delta r))}{\coth(q_1r_g)+\frac{q_2}{q_1}\coth(q_2\delta r)}\right], \tag{S8}$$

where $q_1=\sqrt{\frac{u+\kappa}{D_3}}$, $q_2=\sqrt{u/D_3}$ and $\delta r = r_2 - r_g$.

A Taylor series around $u=0$ then yields

$$j_{\rm bind}(u) \simeq p_r(1-\tau_b u), \tag{S9}$$

and

$$j_{\rm out}(u) \simeq (1-p_r)(1-\tau_e u). \tag{S10}$$

We obtain

$$p_r = 1 - \frac{3\alpha\phi(\gamma)}{\alpha+(\alpha-1)\gamma^2\phi(\gamma)}, \tag{S11}$$

where we introduced $\alpha = r_2/r_g$, $\gamma = r_g\sqrt{\kappa/D_3}$ and the auxiliary function $\phi(\gamma) = (\gamma\coth(\gamma)-1)/\gamma^2$ [1].

The average time it takes for binding reads

$$\begin{aligned}\tau_b = {} & \frac{\alpha}{2\kappa}\left\{5\alpha + \left(4\gamma^2(\alpha-1)-15\alpha\right)\phi(\gamma) + \left(12-15\alpha+2\gamma^2(1-\alpha)^2\right)\gamma^2\phi^2(\gamma)\right\} \\ & \times\left(\alpha+(\alpha-1)\gamma^2\phi(\gamma)\right)^{-1} \\ & \times\left(\alpha+(\gamma^2(\alpha-1)-3\alpha)\phi(\gamma)\right)^{-1}.\end{aligned} \tag{S12}$$

This equation is true for arbitrary values of α. In the main text we explicitly state the case $\alpha=2$. However, in the results section we use $\alpha=\sqrt{23/5}\approx 2.14$, as described in the last section of this SI.

The average time the TF needs for leaving the blob is given by

$$\begin{aligned}\tau_e = {} & \frac{1}{2\kappa}\left\{\alpha(3-\phi^{-1}(\gamma)) + \gamma^2\left((3\alpha-2)\phi(\gamma) + \frac{2+\alpha}{3}(1-\alpha)^2\right) - \frac{\gamma^4}{3}(1-\alpha)^3\phi(\gamma)\right\} \\ & \times(\alpha+(\alpha-1)\gamma^2\phi(\gamma))^{-1}.\end{aligned} \tag{S13}$$

*Electronic address: maximilian.bauer@ph.tum.de
†Electronic address: rmetzler@uni-potsdam.de

B. Target finding probability

To calculate the probability to find the target before dissociating, we consider the one-dimensional diffusion problem

$$\frac{\partial c(z,t)}{\partial t} = D_1 \frac{\partial^2 c(z,t)}{\partial z^2} - k_{\mathrm{off}} c(z,t), \tag{S14}$$

subject to the initial condition $c(z, t = 0) = 1/L$ and the boundary conditions $c(z = 0, t) = 0$ and $\left.\frac{\partial c(z,t)}{\partial z}\right|_{z=L} = 0$. In Laplace space with respect to time we obtain the following solution:

$$c(u,z) = \frac{1}{L(u+k_{\mathrm{off}})}\left(1 - \frac{\cosh((L-z)\sqrt{\frac{u+k_{\mathrm{off}}}{D_1}})}{\cosh(L\sqrt{\frac{u+k_{\mathrm{off}}}{D_1}})}\right) \tag{S15}$$

A Taylor series of $j_{\mathrm{target}}(u) = D_1 \left.\frac{\partial c(z,u)}{\partial z}\right|_{z=0}$ in u yields:

$$j_{\mathrm{target}}(u) \simeq \frac{\tanh(L/\ell)}{L/\ell} + \frac{u}{2k_{\mathrm{off}}}\left(\frac{1}{\cosh^2(L/\ell)} - \frac{\tanh(L/\ell)}{L/\ell}\right), \tag{S16}$$

where $\ell = \sqrt{D_1/k_{\mathrm{off}}}$.

This corresponds to a target finding probability of

$$p_t = \frac{\tanh(L/\ell)}{L/\ell}. \tag{S17}$$

The average time it takes to find the target reads

$$\tau_t = \frac{1}{2k_{\mathrm{off}}}\left(1 - \frac{L/\ell}{\sinh(L/\ell)\cosh(L/\ell)}\right). \tag{S18}$$

II. JUSTIFICATION FOR THE USE OF THE ENSEMBLE AVERAGE

In Figure S1 we plot the ratio of the mean search time for all the eight individual conformations with the mean search time of the corresponding ensemble average at $k_{\mathrm{ass}} = 10^5 \mathrm{M}^{-1}\mathrm{s}^{-1}$. Apparently all the individual curves only scatter about one percent around the value obtained with the ensemble average. Thus it appears appropriate always to use the latter in the main text.

III. DERIVATION OF $\alpha = \sqrt{23/5}$

In principle the parameter α which represents the ratio of the cutoff-radius r_2 and the blob's radius of gyration r_1 is a free parameter which can be used to refine the model. However, in the limit $\kappa \to 0$, that is when no binding to DNA occurs or when there is no DNA present, the escape time τ_e from a blob should coincide with the free diffusion time τ_{3D}. Now using Eq. S13,

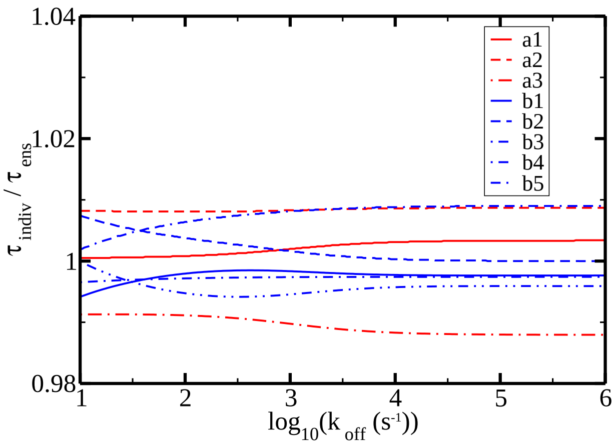

FIG. S1: Ratio of the mean search times obtained with individual conformations with the respective ensemble averaged mean search time at $k_{\mathrm{ass}} = 10^5 \mathrm{M}^{-1}\mathrm{s}^{-1}$.

Equalizing this with $\tau_{3D} = \frac{4r_g^2}{6D_3}$ yields $\alpha = \sqrt{\frac{23}{5}}$. Consequently, this value was chosen in the main text.

[1] Reingruber J, Holcman D (2010) Narrow escape for a stochastically gated brownian ligand. *J Phys Condens Matter* 22:065103.
